# Comparative Study of 4-Aminophenol Removal from Aqueous Solutions by Emulsion Liquid Membranes Using Acid and Basic Type 1 Facilitations. Optimisation and Kinetics

**DOI:** 10.3390/membranes12121213

**Published:** 2022-12-01

**Authors:** Gerardo León, Juliana Otón, Asunción María Hidalgo, María Isabel Saavedra, Beatriz Miguel

**Affiliations:** 1 Departamento de Ingeniería Química y Ambiental, Universidad Politécnica de Cartagena, 30206 Cartagena, Spain; 2 Departamento de Ingeniería Química, Campus de Espinardo, Universidad de Murcia, 30100 Murcia, Spain

**Keywords:** 4-aminophenol, emulsion liquid membranes, acid/basic type 1 facilitations, optimisation, kinetics

## Abstract

The molecule 4-aminophenol (4AP) is recognised as a serious environmental pollutant that enters the environment during the manufacture and processing of a variety of industrial processes and through the degradation of some pharmaceutical products. This paper describes a comparative study of 4AP removal from aqueous solutions by emulsion liquid membranes using acid and basic type 1-facilitated transports. The results are explained by analysing the stripping process through both the different relative acid/basic strength of the hydroxyl and amine groups of the 4AP molecule and the hydrogen-bonding capacity with water of the ionisation products generated by the reaction of 4AP with HCl or NaOH. To optimize the 4AP removal process, the influence of different experimental conditions (stripping agent concentration in the product phase, surfactant concentration in the membrane phase, stirring rate, feed phase/emulsion phase volume ratio, product phase/membrane phase volume ratio and emulsification rate and time) were studied. The kinetics of the removal process has been analysed by fitting the experimental results to first order, second order and the Behnajady and Avrami models. The Behnajady model presents an excellent fit, allowing to calculate both the initial removal rate and the maximal removal conversion. Optimal conditions of the removal process obtained through these parameters are in full agreement with those obtained from the experimental study.

## 1. Introduction

The negative impact of urban industrial effluents on the world’s water resources is a topic of growing concern. Research conducted in the USA showed that 80% of streams contained organic contaminants [1]. Among these organic contaminants, phenols and anilines constitute harmful groups of environmental pollutants due to their toxicity and persistence.

The molecule 4-aminophenol is widely used as a raw material and intermediate in various industries such as dye, rubber, feeding stuffs, petroleum, photography or pharmacy, and it is a metabolite of the emerging pollutant paracetamol and the main component of oxidative hair dye formulations [2,3]. It is toxic and irritable to the eyes, skin and respiratory system [2], causes blood dyscrasias [4] and is known to be a major nephrotoxicant [5,6].

Therefore, careful treatment of wastewater containing 4AP is necessary before discharge into the environment. Different removal technologies have been investigated, such as biodegradation [3], adsorption [7,8,9], electrochemical oxidation [10], enzyme-catalysed hydrogen peroxide oxidation [11] and advanced oxidation processes, including photocatalytic oxidation [12], Fenton [13], photo-Fenton [4], photo-electrochemical oxidation [14], sono-ozonation [15] and sono-photodegradation [16] and solvent-assisted cavitation [17].

Although liquid membrane processes have been used to remove phenol [18,19] and aniline [20,21] from wastewater, no application of these technologies to the removal of 4AP has been previously described.

In a liquid membrane removal process, two miscible liquid phases, the feed and the product phases, are separated by a third phase immiscible in both, the membrane phase. The feed phase contains the component to be removed and the product phase receives that component once it has diffused across the membrane phase. Emulsion liquid membranes are obtained by emulsifying the product phase in the membrane phase and dispersing this emulsion in the feed phase [22]. In order to maximize the removal process, facilitated transport mechanisms have been described. In type 1-facilitated transport, a stripping agent is added to the product phase, which reacts with the component to be removed, yielding a membrane-insoluble product [23].

**Figure 1:**
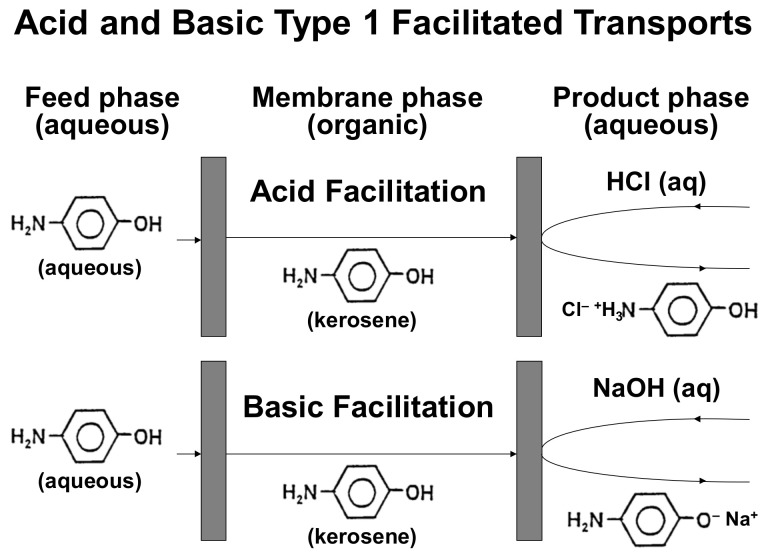
Acid and basic type 1-facilitated transports of 4AP through liquid membranes.

Type 1-facilitated transport has been used to remove phenol and aniline from aqueous solutions using, respectively, basic (NaOH [18]) and acid (HCl [20]) stripping agents in the product phase, yielding sodium phenolate and anilinium chloride, respectively, as insoluble products in the membrane phase. The presence in 4AP of a hydroxyl group and an amino group makes chemical behaviours such as those of phenol or aniline possible. This makes it possibile to remove 4AP using the two kinds of type 1-facilitated transports named: (i) basic type 1-facilitated transport, which uses NaOH as a stripping agent to react with the hydroxyl group of 4AP to form sodium 4-aminophenolate as an insoluble product in the membrane phase (Figure 1); (ii) acid type 1-facilitated transport, which uses HCl as stripping agent to react with the amino group of 4AP to form 4-hydroxyphenylammonium chloride as an insoluble product in the membrane phase (Figure 1).

This paper describes a comparative study of the removal of 4AP from aqueous solutions by emulsion liquid membranes using acid and basic type 1 facilitations. The optimal removal conditions and the kinetic study of the removal process in both facilitations have been carried out analysing the influence of different experimental conditions on the removal of 4AP from the feed phase and fitting the experimental results to different kinetic models, respectively.

Published models that analyse the kinetics of type 1-facilitated transport in emulsion liquid membranes are complex and include a large number of parameters [24,25,26]. Admitting that all the models are approximate and include simplifying assumptions [27], we believe it is interesting to test the fit of the experimental results to simpler mathematical models that may be useful to describe the phenomenon under study. In this sense, in addition to the first-order and second-order kinetic models [28], and considering a possible dependence of the removal rate with the initial 4AP concentration or with the removal time, the Behnajady [29] and Avrami [30] models, widely used in the degradation of organic compounds through advanced oxidation processes and in adsorption processes, respectively, are also studied.

## 2. Materials and Methods

### 2.1. Materials

Analytical grade chemicals were used throughout this study. Kerosene and Span 80 were obtained from Sigma Aldrich (St. Louis, MO, USA), 4-aminophenol (99%) from Alfa Aesar (Ward Hill, MA, USA) and NaOH and HCl (37%) from Panreac (Chicago, IL, USA). Aqueous solutions were prepared in distilled water.

### 2.2. Experimental Procedure

The organic membrane phase was prepared by dissolving the required amount of surfactant (Span 80) in the organic diluent (kerosene). The internal aqueous product phases consisted in aqueous solutions of NaOH or HCl of the required concentration.

A water-in-oil emulsion (primary w/o emulsion) was obtained by mixing the product aqueous phase and the organic membrane phase using a high-speed OMNI MIXER homogeniser (Omni International, Kennesaw, GA, USA). This primary emulsion was gradually added to the external feed phase (aqueous solutions of 4AP 0.15 kg/m^3^, 150 mg/L) contained in a continuously stirred glass cell, using an Agimatic C magnetic stirrer (Selecta, Barcelona, Spain) to adequately disperse the primary emulsion in the feed phase and form the secondary emulsion (w/o/w) that allowed the removal process. The duration of the experiments was 30 min. The external feed aqueous 4AP phase was periodically sampled and, after settling for 3 min to separate the feed and emulsion phases, 2 mL of NaOH 1 M solution was added to 1 mL of the feed sample, measuring the absorbance of the reaction product at a wavelength of 400 nm using an UNICAM UV2 spectrophotometer (Unicam Limited, Cambridge, UK). The extent of the removal process or removal conversion (*X*) was determined by Equation (1), where *C*_0_ and *C_t_* are the initial and time *t* concentrations of 4AP in the feed phase, respectively:

(1) $$X=\frac{C_{0}-C_{t}}{C_{0}}$$

All experiments were performed at room temperature and in duplicate. The results showed a maximum deviation of 5%.

### 2.3. Optimisation of 4-Aminophenol Removal

To optimise the 4-aminophenol removal process by both acid and basic type I facilitations, the effects of different experimental conditions were studied: HCl or NaOH concentration in the product phase (0.010–1.000 M); Span 80 concentration in the membrane phase (0.5–10.0%); feed phase/emulsion phase volume ratio, V_f_/V_e_, (1–8); product phase/membrane phase volume ratio, V_p_/V_m_, (0.75–1.50); emulsification time (2.5–10.0 min) and emulsification rate (1880–3600 rpm) used in the preparation of the primary emulsion; stirring rate (50–300 rpm) of the secondary emulsion (whole feed phase/emulsion phase system).

### 2.4. Kinetic Study

The kinetics of 4-aminophenol removal by emulsion liquid membranes using acid and basic type 1 facilitations were analysed using four kinetic models (first-order, second-order, Behnajady and Avrami models) to determine which best describes the removal process.

(2) $$\ln(1-X)=k_{1}\cdot t$$

where k_1_ (min^−1^) is the rate constant of the first order removal process and *X* is the removal conversion. The value of the rate constant k_1_ can be calculated from the slope of the plot ln(1 − *X*) versus *t*.

The linear form of the second order model [28] is described by Equation (3):

(3) $$\frac{X}{1-X}=C_{0}{\cdot k}_{2}\cdot t$$

where k_2_ (L·mg^−1^·min^−1^) is the rate constant of the second-order removal process. The value of k_2_ can be calculated from the slope of the plot of *X*/(1 − *X*) versus *t*.

The Behnajady model [29] is described by Equation (4):

(4) $$\frac{C_{t}}{C_{0}}=1-\frac{t}{a+b\cdot t}$$

where *a* and *b* are two characteristic constants of the model. This equation can be written in its linearised form:

(5) $$\frac{t}{X}=a+b\cdot t$$

The plot of *t*/*X* versus *t* allows the values of *a* and *b* to be determined from the intercept and the slope, respectively.

The analysis of the extreme cases of Equation (4) allows obtaining the meaning of the two constants of the model.

When the time is very large (it tends to infinity), the inverse of the constant *b* is the maximum removal conversion *C*_min_/*C*_0_ = 1 − 1/*b*, that is, *X*_max_ = 1/*b*, being *C*_min_ and *X*_max_ the minimum 4AP concentration in the feed and the maximum 4AP removal conversion, respectively.

On the other hand, the term *a* can be obtained from the derivative of Equation (4), which is given by d(*C*/*C*_0_)/d*t* = −*a*/(*a* + *b*·*t*)^2^. When the time is very small (approaching zero), the above equation can be written as d(*C*/*C*_0_)/d*t* = −1/*a*. Thus, 1/*a* is the initial rate of the removal process (V_0_).

The linear form of the Avrami kinetic model [30] is usually described by Equation (5). The values of the model constants *K_av_* (Avrami constant rate) and *n_av_* (Avrami order can be obtained from the plot of ln[−ln(1 − *X*)] versus ln*t*:

(6) $$\ln\left[-\ln\left(1-X\right)\right]=\ln K_{av}+n_{av}\cdot \ln t$$

## 3. Results and Discussion

### 3.1. Optimisation of 4-Aminophenol Removal Removal Using Acid and Basic Type 1 Facilitations

**Figure 2:**
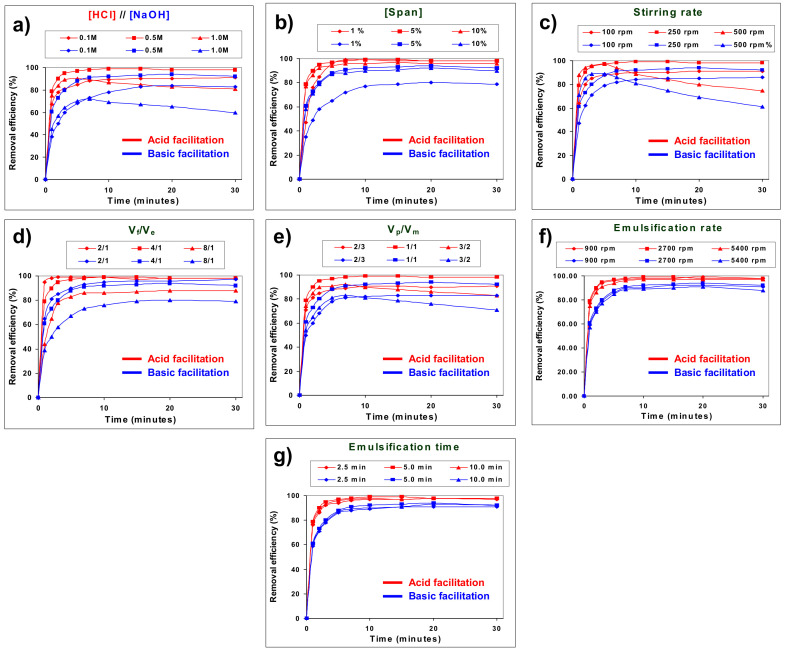
Effect of different experimental conditions on the removal efficiency of 4-aminophenol from the feed phase by acid and basic type 1 facilitations.

Figure 2a–g shows the effects of different experimental conditions on the removal efficiency (100·*X*) of 4-aminophenol from the feed phase by acid and basic type 1 facilitations. No emulsion breakage was observed under the tested experimental conditions, except with higher HCl and NaOH concentrations and higher stirring speed.

Figure 2a shows the effect of the stripping agent concentration in the permeate phase on the removal efficiency of 4AP. It can be seen that in both type 1 facilitations, 4AP removal increases with increasing striping agent concentration from 0.1 to 0.5 M. Increasing the concentration of H^+^ or OH^−^ in the product phase increases their reaction with the amino or hydroxyl groups of 4AP, respectively, which favours the transport of 4AP to the product phase [31]. A further increase in the stripping agent concentration up to 1.0 M leads to a decrease in 4AP removal due to both the membrane swelling caused by the increased ionic strength difference between the internal and external phases and the partial hydrolysis of the ester bonds of Span 80 (especially in the case of basic facilitation), which involves a reduction of its surfactant properties and, consequently, some emulsion destabilisation [32].

The effect of surfactant concentration on 4AP removal efficiency is shown in Figure 2b. As can be seen, in both type 1 facilitations, the removal of 4AP increases as the Span 80 concentration in the membrane phase increases from 0.5% to 5.0% due to a decrease in the interfacial tension between the phases that favours the formation of smaller and more stable emulsion globules, resulting in a larger mass transfer surface [33]. Increasing the surfactant concentration up to 10% leads to an increase in viscosity and membrane thickness that negatively influences 4AP transport by decreasing the diffusion and mass transfer coefficient [34]; this counteracts the effect of lowering interfacial tension and results in a slight decrease in 4AP removal.

The effect of the stirring speed, used to mix the primary emulsion and feed solution, on the removal efficiency of 4AP is shown in Figure 2c. It was found that in both type 1 facilitations, the removal of 4AP increases with increasing stirring speed from 100 to 250 rpm. Increasing the stirring speed leads to the reduction of the emulsion droplets’ size and thickness, thus providing a higher interfacial contact area between the feed and membrane phases and increasing mass transfer [35]. A further increase in stirring speed leads to a decrease in 4AP removal due to the breakdown of emulsion globules by impact with the impeller, cell wall and other emulsion globules [36].

The effect of the feed phase/emulsion phase volume ratio (V_f_/V_e_) on the removal efficiency of 4AP was studied at a constant permeate phase/membrane phase volume ratio by decreasing the volume of the feed aqueous phase while keeping the volume of the emulsion phase (membrane phase plus product phase) constant. As can be seen in Figure 2d, in both type 1 facilitations the removal of 4AP significantly increases when the ratio decreases from 8 to 2. The decrease in the external feed phase volume (decrease in the ratio) causes an increase in both the amount of available emulsion globules per total amount of 4AP in the feed phase (increase in the membrane area per total volume of feed) and the amount of stripping agent per total amount of 4AP in the feed phase, leading to an increase in 4AP removal [37].

The effect of the product phase/membrane phase volume ratio (V_p_/V_m_) on the removal efficiency of 4AP was studied at a constant feed phase/emulsion phase volume ratio by increasing the volume of the permeate aqueous phase while keeping constant the volume of the membrane phase. The results show (Figure 2e) that in both type 1 facilitations, the removal of 4AP increases as this ratio increases up to 1, but it decreases at higher values. The increase in the volume of the product aqueous phase leads to a decrease in the thickness of the membrane phase [38] and an increase in the amount of stripping agent in the product phase at a constant amount of 4AP in the feed phase, all of which leads to an increase in the removal efficiency. On the other hand, it also leads to an increase in the viscosity of the emulsion phase, producing an increase in the size of the emulsion droplets and, consequently, a decrease in the mass transfer surface area leading to a decrease in the removal efficiency [39]. The overall result of these two opposing effects was an increase in 4AP removal as the V_p_/V_m_ ratio increased to 1, but a decrease at higher V_p_/V_m_ ratios.

Figure 2f,g show that, for both type 1 facilitations, neither the emulsification time nor the emulsification speed have a significant effect on the removal efficiency of 4AP. As no significant conclusion is expected from the kinetic study of these two parameters, they will not be included in this kinetic study.

According to the results described above, the optimal experimental conditions are 0.5 M stripping agent concentration (HCl or NaOH) in the permeate phase, 5% surfactant concentration in the membrane phase, 250 rpm stirring rate of secondary emulsion, 2/1 V_f_/V_e_ ratio, 1/1 V_p_/V_m_ ratio, 5 min emulsification time and 2700 rpm emulsification rate. These were the standard conditions used in all the experiments described above, except in the case of the treatment ratio where a 4/1 ratio was used, with a view to a more economical profitability of the removal process. At the optimal conditions, the maximal removal conversions for acid and basic facilitations were 0.99 and 0.95, respectively, at 10 min.

### 3.2. Comparison of Acid and Basic Type 1-Facilitated Transports of 4-Aminophenol

In all experimental conditions studied, acid type 1-facilitated transport leads to a faster and higher 4AP removal than basic type 1-facilitated transport (Figure 2). These differences must be a consequence of the different behaviour of the 4AP molecule in acid and basic stripping reactions, due both to the different reactivity (different relative acid/basic strength) of the amine and hydroxyl functional groups towards the stripping agents and to the different capacity to form hydrogen bonds with water of the products generated at the membrane/product interface by the reaction of 4AP with HCl or NaOH (4-hydroxyphenyl ammonium and 4-aminophenolate, respectively) (Figure 1) [40,41].

Both the –OH and –NH_2_ groups have a +R electronic resonance effect. This means that both groups donate π electronic density to the aromatic ring and then to the other functional group located in position 4 of the benzene ring. The +R effect of –NH_2_ increases the negative charge density of the –OH group, decreasing its acid character (accepts electronic density, according to the Lewis acid–base theory). The +R effect of –OH increases the negative charge density of the –NH_2_ group, leading to an increase in its basic character (gives electronic density, according to the Lewis acid–base theory). Consequently, a higher relative basic strength of the –NH_2_ group of 4-aminophenol can be expected compared to the relative acid strength of the –OH group. This should lead to an easier removal of 4NP from aqueous solution by acid facilitation (HCl in the product phase) than by basic facilitation (NaOH in the product phase).

In addition, the water solubility effects of the acid and basic reaction products generated in the membrane/product interface must be also considered. The product of the reaction of 4-aminophenol with NaOH is sodium 4-aminophenolate, an ionic compound with two hydrogens with the capability to form hydrogen bonds with water (the two hydrogens of the –NH_2_ group). The product of the reaction of 4-aminophenol with HCl is 4-hydroxyphenylammonium chloride, also an ionic compound but with four hydrogens with the capability to form hydrogen bonds (the hydrogen of the –OH group and the three hydrogens of the –NH_3_^+^ group). This enhanced hydrogen-bonding capacity should improve 4-aminophenol transport from the membrane to the product phase in the case of acid type 1 facilitation, which should lead to an increase in the rate and efficiency of the removal process.

### 3.3. 4-Aminophenol Removal Kinetics

**Figure 3:**
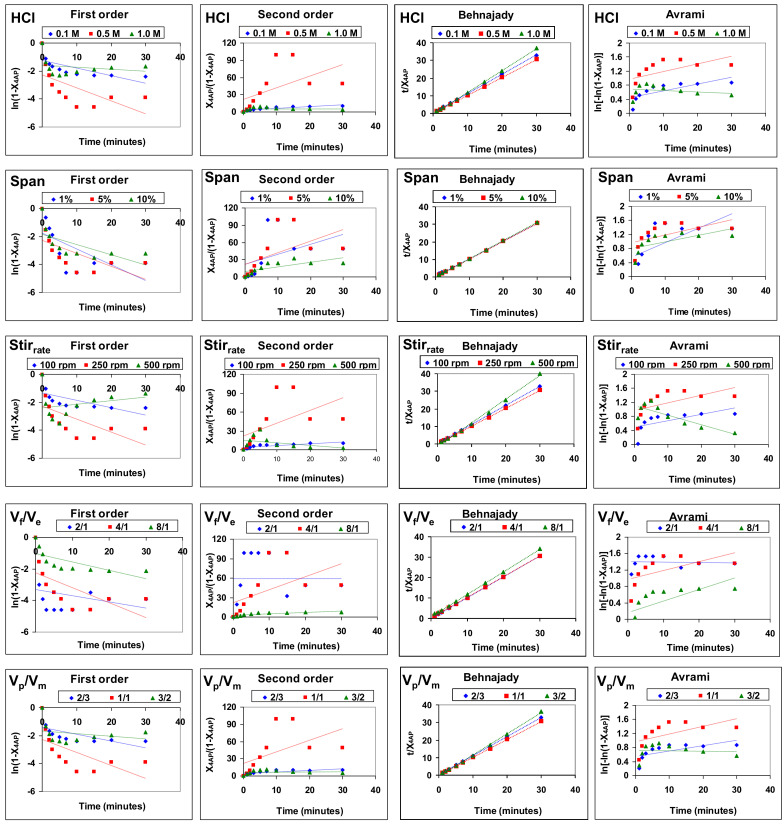
Fitting of the results of 4AP removal by emulsion liquid membranes using acid type 1 facilitation to the four kinetic models studied under the different experimental conditions.

**Figure 4:**
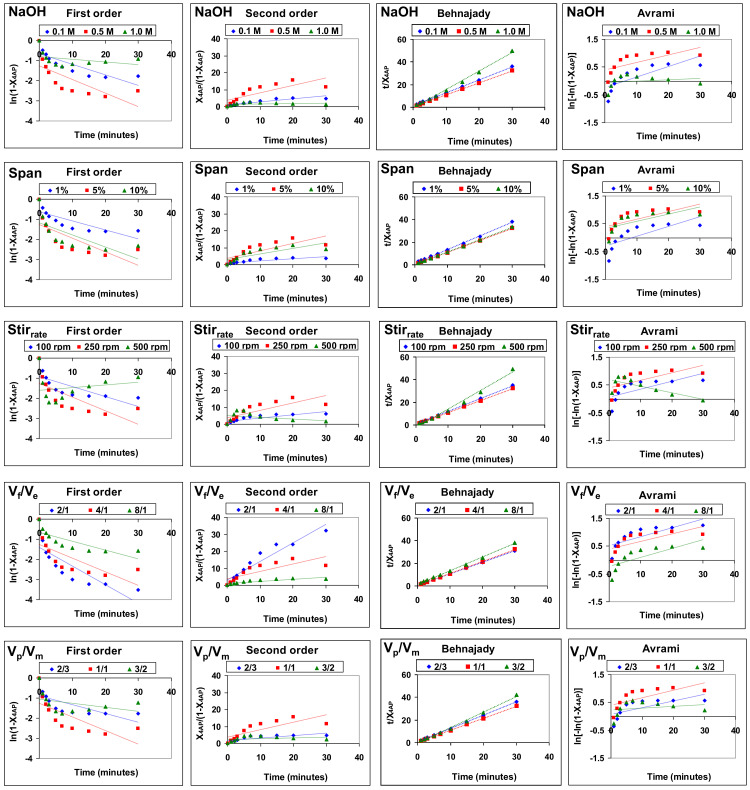
Fitting of the results of 4AP removal by emulsion liquid membranes using basic type 1 facilitation to the four kinetic models studied under the different experimental conditions.

The fit of the data of 4AP removal by emulsion liquid membranes using acid and basic type 1 facilitations to the four models described above, under the different experimental conditions studied, is shown in Figure 3 and Figure 4, respectively. The reliability of the fit was determined based on the values of the determination coefficient (R^2^), as shown in Table 1.

The experimental data of 4-aminophenol removal best fit the Behnajady model, which showed determination coefficient values higher than 0.988 (0.9883–1.0000) for all the experimental conditions studied. The first-order (0.0408–0.4998), second-order (0.0006–0.7335) and Avrami (0.0034–0.7028) models provided much lower determination coefficients.

The values of the Behnajady kinetic constants (*a* and *b*), and of the initial removal rate (V_0_) and maximal removal conversion (*X*_max_) parameters calculated from those kinetic constants, are shown in Table 2.

The analysis of the values of these parameters (V_0_ and *X*_max_) in the different experimental conditions studied confirms both the higher and faster 4AP removal by acid type 1 facilitation than by basic type 1 facilitation as well as the optimal removal conditions described above. The use of the acid type 1-facilitated transport mechanism leads to maximum removal conversion values and initial removal rates that are 20–40% and 15–25%, respectively, higher than those obtained using the basic type 1-facilitated transport mechanism.

In both facilitations, anomalous results are only obtained at the higher stirring rate, where there is a significant emulsion breakage that leads to negative values of the constant “*a*” and, consequently, to negative values of V_0_.

At standard removal conditions, the relationship between the values of the experimental removal conversion and those obtained from the Behnajady model shows determination coefficients of 0.991 and 0.990 for acid and basic facilitations, respectively.

All this allows us to affirm that Behnajady’s model satisfactorily describes the kinetics of the removal of 4AP from its aqueous solutions by emulsion liquid membranes using both acid and basic type 1 facilitations.

## 4. Conclusions

This paper describes a comparative study of the removal of 4-aminophenol from aqueous solutions by emulsion liquid membranes using acid and basic type 1-facilitated transports, analysing the effect of different experimental conditions on the removal process in order to optimise it, and studying its kinetics by fitting the experimental results to four kinetic models (first-order, second-order, Behnajady and Avrami models).

Acid type 1-facilitated transport leads to higher and faster 4AP removal than basic type 1-facilitated transport in all the experimental conditions studied. This result has been explained by analysing the stripping process through both the different relative acid/basic strength of the hydroxyl and amine groups of the 4AP molecule and the hydrogen-bonding capacity with water of the ionisation products generated by the reaction of 4AP with HCl or with NaOH.

Optimisation of the removal process shows that the optimal 4AP removal conditions are: 0.5 M stripping agent concentration (HCl or NaOH) in the product phase, 5% Span 80 concentration in the membrane phase, 250 rpm stirring rate of secondary emulsion, 2/1 V_f_/V_e_ ratio, 1/1 V_p_/V_m_ ratio, 5 min emulsification time and 2700 rpm emulsification rate.

The experimental data for 4AP removal best fit the Behnajady model, which showed determination coefficient values ranging from 0.9883 to 1.0000. The values of the initial removal rate (V_0_) and maximal removal conversion (*X*_max_) calculated from the Behnajady model kinetic constants, under the different experimental conditions studied, are in full agreement with both the optimal removal conditions obtained experimentally and the higher (20–40%) and faster (15 to 25%) 4AP removal of acid type 1 facilitation over basic type 1 facilitation.

## Figures and Tables

**Table 1:** Determination coefficients (R^2^) of the fit of the different experimental parameters to the studied kinetic models in the removal of 4AP by ELM using acid and basic type 1 facilitations.

Acid Type 1 Facilitation						Basic Type 1 Facilitation					
Parameter/Value		First Order	Second Order	Behnajady	Avrami	Parameter/Value		First Order	Second Order	Behnajady	Avrami
[HCl]	0.1 M	0.4911	0.7335	1.0000	0.5641	[HCl]	0.1 M	0.6871	0.8072	0.9992	0.6190
	0.5 M	0.3873	0.2958	0.9999	0.3466		0.5 M	0.5160	0.6315	0.9995	0.4845
	1.0 M	0.0408	0.0006	0.9990	0.0472		1.0 M	0.1249	0.0913	0.9943	0.0365
[Span 80]	1%	0.4265	0.2001	0.9999	0.3540	[Span 80]	1 %	0.6274	0.7368	0.9990	0.5569
	5%	0.3873	0.2958	0.9999	0.3466		5 %	0.5160	0.6315	0.9995	0.4845
	10%	0.4119	0.5281	0.9999	0.4079		10 %	0.4970	0.6252	0.9995	0.4633
Stir_rate_	100 rpm	0.4224	0.6262	0.9999	0.4127	Stir_rate_	100 rpm	0.5444	0.70386	0.9998	0.4928
	250 rpm	0.3873	0.2958	0.9999	0.3466		250 rpm	0.5160	0.6315	0.9995	0.4845
	500 rpm	0.0606	0.1611	0.9959	0.7028		500 rpm	0.0342	0.1240	0.9883	0.5758
V_f_/V_emul_	2/1	0.0738	0.0001	0.9999	0.0034	V_f_/V_emul_	2/1	0.6540	0.9250	1.0000	0.6125
	4/1	0.3873	0.2958	0.9999	0.3466		4/1	0.5160	0.6315	0.9995	0.4845
	8/1	0.4998	0.6572	0.9995	0.4036		8/1	0.6171	0.7364	0.9993	0.5730
V_m_/V_p_	2/3	0.3898	0.5691	0.9999	0.4446	V_m_/V_p_	2/3	0.4968	0.6161	0.9996	0.4792
	1/1	0.3873	0.2958	0.9999	0.3466		1/1	0.5160	0.6315	0.9995	0.4845
	3/2	0.0589	0.0058	0.9986	0.0043		3/2	0.1387	0.0852	0.9950	0.0571

**Table 2:** Behnajady kinetic constants (*a*, *b*), their derived parameters initial removal rate (V_0_) and maximal removal conversion (*X*_max_).

Acid type 1 Facilitation						Basic Type 1 Facilitation					
Parameter/Value		Parameter *a*	V_0_ (1/*a*)	Parameter *b*	*X*_max_ (1*/b*)	Parameter/Value		Parameter *a*	V_0_ (1/*a*)	Parameter *b*	*X*_max_ (1/*b*)
[HCl]	0.1 M	0.4076	2.4534	1.0862	0.9206	[HCl]	0.1 M	1.5158	0.6597	1.1374	0.8792
	0.5 M	0.1107	9.0334	1.0131	0.9871		0.5 M	0.4240	2.3585	1.0559	0.9435
	1.0 M	0.4290	2.3310	1.2327	0.8112		1.0 M	0.7368	1.3572	1.6297	0.6136
[Span 80]	1%	0.1125	8.8889	1.0141	0.9861	[Span 80]	1%	1.4534	0.6880	1.1198	0.8349
	5%	0.1107	9.0334	1.0131	0.9871		5%	0.4240	2.3585	1.0559	0.9435
	10%	0.1578	6.3371	1.0327	0.9683		10%	0.4442	2.2512	1.0829	0.9234
Stir_rate_	100 rpm	0.3129	3.1959	1.0865	0.9204	Stir_rate_	100 rpm	0.7832	1.2678	1.1329	0.8827
	250 rpm	0.1107	9.0334	1.0131	0.9871		250 rpm	0.4240	2.3585	1.0559	0.9435
	500 rpm	−1.2361	-	1.3356	0.7487		500 rpm	−2.1923	-	1.6251	0.6153
V_f_/V_emul_	2/1	0.0242	41.3223	1.0021	0.9979	V_f_/V_emul_	2/1	0.4565	2.1906	1.0153	0.9849
	4/1	0.1107	9.0334	1.0131	0.9871		4/1	0.4240	2.3585	1.0559	0.9435
	8/1	0.6780	1.4749	1.1069	0.9034		8/1	1.3214	0.7456	0.2945	0.830.
V_m_/V_p_	2/3	0.2410	1.0908	1.0908	0.9168	V_m_/V_p_	2/3	0.6886	1.4522	1.1732	0.8524
	1/1	0.1107	9.0334	1.0131	0.9871		1/1	0.4240	2.3585	1.0559	0.9435
	3/2	0.4028	2.4826	1.1970	0.8354		3/2	0.5052	1.9794	1.3774	0.7260

## Data Availability

Not applicable.
